# The Masters athlete in Olympic weightlifting: Training, lifestyle, health challenges, and gender differences

**DOI:** 10.1371/journal.pone.0243652

**Published:** 2020-12-04

**Authors:** Marianne Huebner, David Meltzer, Wenjuan Ma, Holly Arrow

**Affiliations:** 1 Department of Statistics and Probability, Michigan State University, East Lansing, MI, United States of America; 2 Center for Statistical Training and Consulting, Michigan State University, East Lansing, MI, United States of America; 3 College of Integrative Sciences and Arts, Arizona State University, Mesa, AZ, United States of America; 4 Department of Psychology, University of Oregon, Eugene, OR, United States of America; University of Rome, ITALY

## Abstract

**Background:**

Olympic weightlifting requires strength, speed, and explosive power. Vigorous physical activity such as Olympic weightlifting, for older adults has many benefits from improved strength, social interactions, and a healthy and independent lifestyle. Little is known about the training habits, health, and lifestyle of Masters weightlifters that includes top level athletes as well as beginners, and there is a dearth of data on women.

**Objectives:**

The primary aim was to describe demographics, training habits, and health including prevalence of injury and chronic disease in male and female Masters athletes in Olympic weightlifting. The secondary aim was to study gender differences and the age and impact of menopause on participation in the sport.

**Results:**

The 958 participants (46% men), ages 34 to 87, mostly train 3 to 4 days per week in 1 to 2 hour sessions. This is a highly educated and affluent group, 84% are white, 72% are married, 85% are post-secondary graduates. Exercise can also increase the risk of injury compared to less active older adults, but the rates of injury in weightlifting affecting training are lower than seen in other sports. The prevalence of depression and mental health is similar to a general population. Stress levels and sleep disturbances are more common among women than men. Women reach menopause at a similar age as women in industrialized countries, but menopausal symptoms constrained the training.

**Conclusion:**

Older athletes are capable of rigorous training programs and top performances while adjusting to changes due to biological aging. Weightlifting athletes, coaches, and health professionals must be aware of patterns of injuries and gender differences to incorporate successful prevention strategies. Knowledge of presentations of menopause and impact of menopausal symptoms on training allows women and health care providers to make informed treatment decisions.

## Introduction

Exercise has a large impact on preservation of muscle power and cellular function and on maintaining an active independent lifestyle for older adults [1, 2]. Olympic-style weightlifting, for example, requires strength, speed, and explosive power [3, 4]. Individuals differ widely in how they age and in how they adapt to an exercise program [5]. Several recommendations have been published by the American College of Sports Medicine (ACSM) on quantity and quality of exercise for optimal cardiorespiratory and muscular fitness [6]. However, despite the recognized benefits of exercise, physical and mental health or lifestyle demands may present obstacles to regular maintenance of an exercise regimen. Masters weightlifters are aged 35 and up, and age-associated performance decline for these has been studied for Masters weightlifters performing at high level competitions [7–11]. However, these studies were based on data from World Championships or world records and thus may not apply directly to a broader population including athletes at the local or more recreational level. Our study seeks to expand these investigations to that broader population.

While exercise cannot stop the biological aging process, a rigorous training regime may lead to slower declines in strength or aerobic capacity from their maximal values [1]. However, faulty training strategies can lead to overuse injuries which can be a major detriment to continuing the training regime [12]. Athletes and coaches should thus be made aware of patterns of injuries for older ages to inform their design of prevention strategies. Data regarding the manner in which this older population is able to engage in intense physical activities, despite frequent health challenges arising from chronic disease or injuries, can inform sport scientists and medical health professionals and help guide their recommendations. This type of information is particularly important for sports that have undergone rapid growth in popularity among older adults, since such growth inevitably attracts a vast new population of individuals who are at relatively high risk for injury. Olympic-style weightlifting is one such sport, for its worldwide popularity among older adults has exploded in recent years due to the CrossFit phenomenon. Participation in Masters weightlifting for individuals age 35 and up has grown rapidly, with women’s participation in particular increasing by roughly an order of magnitude over the past decade. The dramatic recent increase has come most often from those without prior experience in weightlifting. The participation rate in the USA National Masters Weightlifting Championships has changed from 2015 with 244 competitors (44.4% female and 55.6% male) to 718 in 2019 (with 58.7% female and 41.3% male). Over a ten-year period, from 2009 to 2019, the proportion of women competitors in this event climbed from 14% to 59%. Some Masters athletes are experienced in competitions and continue their athletic pursuit without any lengthy breaks, while many others return to sport participation after extended periods of inactivity, or they train sporadically [1]. An overall reduction in the exercise training stimulus (i.e. exercise-training intensity, session duration and weekly frequency) with advancing age is associated with a reduction in endurance exercise performance. However, a causal link is not clear, since many factors underlie the decreasing ability to maintain exercise intensity and volume with increasing age [13]. We seek to shed light on this issue by examining a wide range of factors that may affect performance of Masters weightlifters.

There is a dearth of data on training habits and health challenges for older women participating, recreationally or competitively, in sports. Gender disparities have been noted for sports injuries [14] and for psychological factors reported in a general population [15]. The performance decline in Olympic weightlifting is steeper for women than for men during the transition to menopause [7]. It has also been shown that regular physical activities can mitigate adverse changes in women due to lifestyle factors or hormonal changes [16, 17]. Our study provides a unique opportunity to observe gender differences in training programs and health factors, and study age and impact of menopause on training in a weightlifting population.

The primary aim of this study was to describe demographics, training habits, and physical health including prevalence of injury and chronic disease in male and female Masters athletes in Olympic weightlifting. The secondary aim was to study gender differences and the age and impact of menopause on participation in the sport.

## Materials and methods

The “Masters” category in weightlifting comprises all lifters who turn age 35 or older any time during the current calendar year, thus including individuals whose current chronological age is 34. All 3216 Masters weightlifters registered with USA Weightlifting (USAW) during 2020 were invited to participate in an online survey. This group includes all those who turn age 35 or older between January 1 and December 31 of 2020; it accounts for about 12% of the USAW membership. The questionnaire was created and hosted in Qualtrics software [18] and was piloted by sending it to 10 USAW Masters members. Feedback on wording and choice of questions was incorporated. The invitation was sent via USAW’s email system in January 2020 with a reminder two weeks later. To increase participation rates, a notification with a link to the survey was also posted on Masters weightlifting Facebook sites. The survey was constructed to collect information on demographics, physical activities, training habits, health and injuries, as well as free form comments about the impact of weightlifting on their lives. Consent was obtained and the study was approved by the Institutional Review Board of Michigan State University (Study No. 00003824).

### Statistical analysis

Survey responses were checked for duplicates and internal consistency regarding age, gender, and dates. Responses with missing birth dates or gender were removed. Continuous variables were summarized with median and ranges, and categorical variables were summarized with frequencies and percentages, stratified by gender and age groups. Age groups were defined as 35 to 44 years, 45 to 59 years, and 60 years and older. The age groupings were chosen, first, to separate the age group with the largest recent increase in participation (the youngest group), second, to focus on a middle age group that represents ages of transition to menopause (45–59), and finally, to study a combined older age group covering a wide age range, due to relative paucity of data for older ages. Chi-square tests were used to test differences in categorical variables between women and men. A p-value of 0.05 was considered statistically significant. A more detailed investigation of the shoulder issues was carried out due to their relative prevalence. A subgroup analysis of training habits of male athletes with 20 or more years of experience was added as it emerged that this was a distinct cohort for men.

Cumulative incidence functions were used to describe occurrences (menopause, injuries) by age. Cumulative incidence functions at a specific age are defined as the probability that an event occurs before that age. Cox proportional hazard models were used to estimate the effect of gender [19].

Answers to two open-ended questions regarding the influence of weightlifting on life or vice versa were coded according to themes, and a random sample was checked for reliability. The analyses were performed using the statistical software R version 3.6.1 [20] and the packages ggplot2_3.3.0 and survival_2.44–1.1. The study was reported according to the STROBE statement [21].

## Results

**Fig 1:**
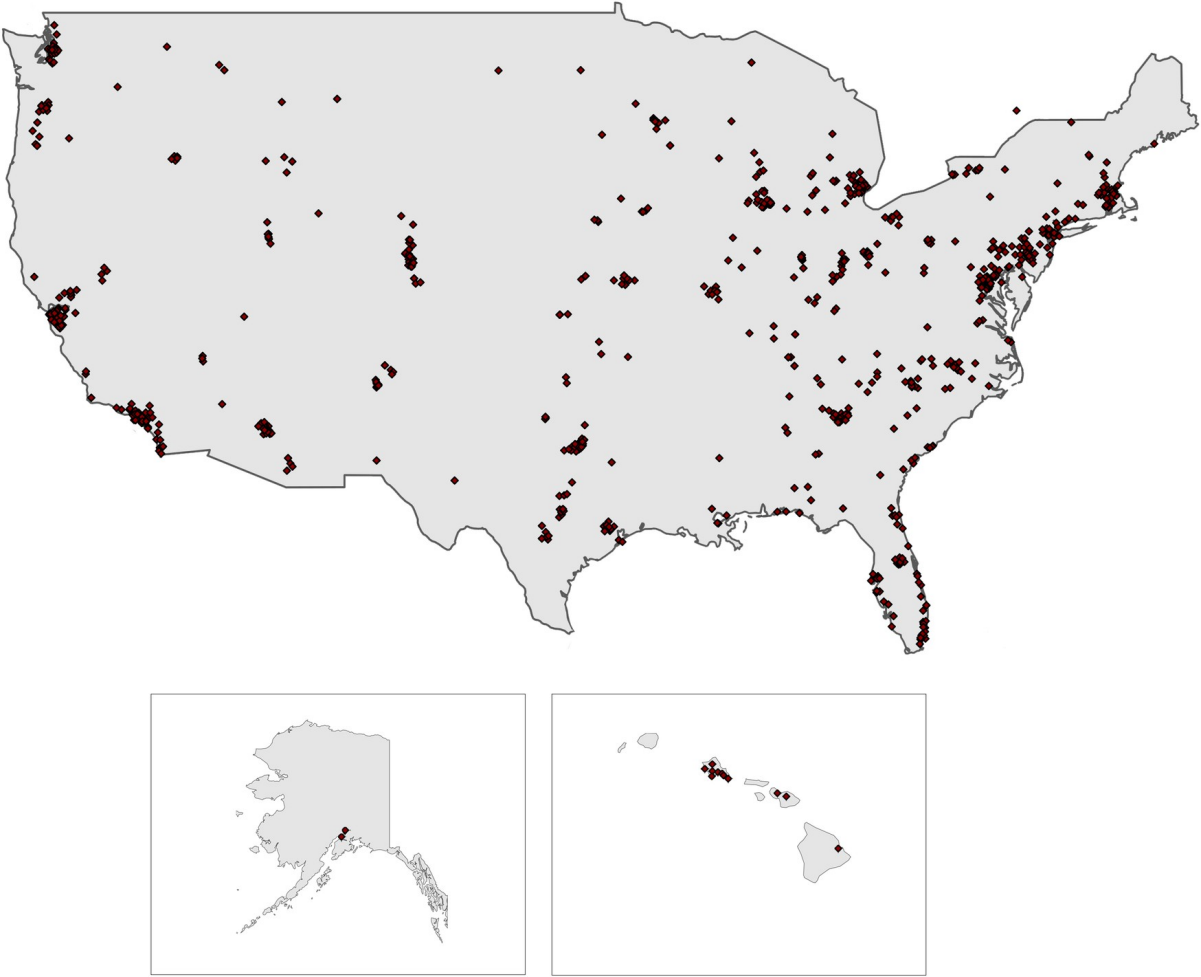
Responses by geographic location USA map.

The response rate was 30.6% for the entire group of Masters weightlifters registered with the national governing body of Olympic weightlifting, USA Weightlifting. USAW membership is required for competitive athletes, coaches, and referees. Responses were from across the United States and 8 from overseas (Fig 1). A total of 958 responses were included in the analysis with women outnumbering men (54.4%) to (45.6%) (S1 Fig). This excluded one participant who identified as “transgender” and two as “other.” In addition to responding about training habits and physical exercise, 947 participants also answered questions about impact of health, work, and psychological factors on training, and 435 provided comments on the influence of weightlifting training on their life and vice versa.

### Demographics

The median age of participants was 45 (range 34 to 87), but strongly skewed towards the younger ages. Only 19.2% of women and 28.4% of men were aged 55 or older. Although 8% of the men were aged 70 or older, only 1% of women are in that same age category. The majority 71.6% was married. This was a highly educated and affluent group in which 47.7% attended graduate school, 60.7% had a household income of $100K or above, and 80.4% were employed full time. Most were white/Caucasian (83.9%) (Table 1).

**Table 1:** Demographics by gender.

	Women (n = 521)	Men (n = 437)	Total (n = 958)
**Age**, median (range)	44 (34–75)	46 (34–87)	45 (34–87)
**Age groups,** n(%)			
35–39	159 (30.5%)	116 (26.5%)	275 (28.7%)
40–44	111 (21.3%)	68 (15.6%)	179 (18.7%)
45–49	74 (14.2%)	79 (18.1%)	153 (16.0%)
50–54	77 (14.8%)	50 (11.4%)	127 (13.3%)
55–59	49 (9.4%)	39 (8.9%)	88 (9.2%)
60–64	35 (6.7%)	29 (6.6%)	64 (6.7%)
65–69	11 (2.1%)	22 (5.0%)	33 (3.4%)
70–74	4 (0.8%)	18 (4.1%)	22 (2.3%)
75–79	1 (0.2%)	10 (2.3%)	11 (1.1%)
80+	0 (0.0%)	6 (1.4%)	6 (0.6%)
**Marital Status,** n(%)			
Married	339 (65.6%)	343 (78.7%)	682 (71.6%)
Divorced/widowed/separated	74 (14.4%)	35 (8.0%)	109 (11.4%)
Not married	104 (20.1%)	58 (13.3%)	162 (17.0%)
missing	4	1	5
**Race,** n(%)			
White/Caucasian	425 (82.5%)	369 (85.6%)	794 (83.9%)
Black or African American	19 (3.7%)	10 (2.3%)	29 (3.1%)
Asian	25 (4.9%)	20 (4.6%)	45 (4.8%)
Native Hawaiian or Pacific Islander	6 (1.2%)	5 (1.2%)	11 (1.2%)
American Indian or Alaska Native	1 (0.2%)	1 (0.2%)	2 (0.2%)
Multiple + other	39 (7.5%)	26 (5.9%)	65 (6.8%)
Missing/Decline to answer	6	6	12
**Hispanic**, yes (%)	46 (8.9%)	34 (7.8%)	80 (8.4%)
Missing/Decline to answer	3	2	5
**Income,** n(%)			
<$100,000	42 (8.1%)	31 (7.1%)	73 (7.6%)
$100,000-$149,000	110 (22.5%)	110 (26.3%)	220 (24.3%)
$150,000 - $200,000	73 (14.9%)	71 (17.0%)	144 (15.9%)
>$200,000	99 (20.2%)	87 (20.8%)	186 (20.5%)
Missing/Decline to answer	32	19	51
**Employment,** n(%)			
Full-time	409 (78.7%)	359 (82.5%)	768 (80.4%)
Part-time	67 (12.9%)	26 (6.0%)	93 (9.7%)
Retired	28 (5.4%)	41 (9.4%)	69 (7.2%)
Unemployed	16 (3.1%)	9 (2.1%)	25 (2.6%)
Missing/Decline to answer	1	2	3
**Education,** n(%)			
High school/GED or less	14 (2.6%)	17 (3.9%)	31 (3.2%)
Some college	64 (12.3%)	79 (18.1%)	143 (14.9%)
College degree	165 (31.7%)	162 (37.1%)	327 (34.1%)
Some graduate school	35 (6.7%)	20 (4.6%)	55 (5.7%)
Graduate school	243 (46.6%)	159 (36.4%)	402 (42.0%)

### Weightlifting training habits

#### Duration of training session

For both men and women, training session duration of 1–2 hours was most common, for every age category. In the 60+ age category this was reported by 61.2% of the men and by 78.4% of women; at other ages between 69–78% reported that same 1–2 hours duration. Among the women (but not the men), the proportion reporting durations *over* 2 hours fell with increasing age (from 19.3% age 35–44, to 11.8% age 60+). The proportion reporting durations *less* than 1 hour increased from 9.8% (age 35–44) to 23.5% (age 60+) for men, but that same pattern was not evident among the women (Table 2). Training times up to one hour were reported by 27.1% of these more experienced older men (i.e. with more than 5 years of competition experience), versus 18.1% for those of the same age with less experience. The majority of experienced athletes in this age group design their own training program, 68.8% compared to 21.2%.

**Table 2:** Training habits for women and men by age group.

	Women				Men			
	Age 35–44	Age 45–59	Age 60+	Total	Age 35–44	Age 45–59	Age 60+	Total
	N = 270	N = 200	N = 51	N = 521	N = 184	N = 168	N = 85	N = 437
**Training program,** n(%)								
Coach	240 (88.9%)	170 (85.0%)	50 (98.0%)	460 (88.3%)	126 (68.5%)	109 (64.9%)	31 (36.5%)	266 (60.9%)
Own program	18 (6.7%)	20 (10.0%)	1 (2.0%)	39 (7.5%)	42 (22.8%)	49 (29.2%)	40 (47.1%)	131 (30.0%)
Book/website	16 (5.9%)	8 (4.0%)	0 (0.0%)	24 (4.6%)	18 (9.8%)	8 (4.8%)	7 (8.2%)	33 (7.6%)
subscription	17 (6.3%)	11 (5.5%)	0 (0.0%)	28 (5.4%)	22 (12.0%)	10 (6.0%)	1 (1.2%)	33 (7.6%)
No system	4 (1.5%)	3 (1.5%)	0 (0.0%)	7 (1.3%)	10 (5.4%)	4 (2.4%)	7 (8.2%)	21 (4.8%)
Combination 2+	17 (6.3%)	11 (5.5%)	0 (0.0%)	28 (5.4%)	26 (14.1%)	12 (7.1%)	1 (1.2%)	39 (8.9%)
**Training days,** n(%)								
1–2 days	8 (3.0%)	10 (5.0%)	7 (13.5%)	25 (4.8%)	6 (3.3%)	9 (5.4%)	14 (16.5%)	29 (6.6%)
3 days	42 (15.6%)	45 (22.5%)	22 (43.1%)	109 (20.9%)	37 (20.1%)	58 (34.5%)	40 (47.1%)	135 (30.9%)
4 days	87 (32.2%)	77 (38.5%)	8 (15.7%)	172 (33.0%)	72 (39.1%)	59 (35.1%)	20 (23.5%)	151 (34.6%)
5 days	108 (40.0%)	52 (26.0%)	12 (23.5%)	172 (33.0%)	55 (29.9%)	32 (19.0%)	6 (7.1%)	93 (21.3%)
6–7 days	23 (8.5%)	15 (7.5%)	2 (3.9%)	40 (7.7%)	14 (7.6%)	8 (4.8%)	4 (4.7%)	26 (5.9%)
**Training time,** n(%)								
Up to 1 hour	20 (7.4%)	28 (14.0%)	5 (9.8%)	53 (10.2%)	18 (9.8%)	25 (15.0%)	20 (23.5%)	63 (14.4%)
1–1.5 hours	87 (32.3%)	62 (31.0%)	18 (35.3%)	167 (32.1%)	66 (35.9%)	64 (38.3%)	27 (31.8%)	157 (36.0%)
1.5–2 hours	110 (40.9%)	75 (37.5%)	22 (43.1%)	207 (39.8%)	76 (41.3%)	53 (31.7%)	25 (29.4%)	154 (35.3%)
>2 hours	52 (19.3%)	35 (17.5%)	6 (11.8%)	93 (17.9%)	24 (13.0%)	25 (15.0%)	13 (15.3%)	62 (14.2%)
Missing	1	0	0	1	0	1	0	1
**Training location,** n(%)								
WL gym	128 (47.4%)	94 (47.0%)	27 (52.9%)	249 (47.8%)	93 (50.5%)	74 (44.0%)	33 (38.8%)	200 (45.8%)
CF gym	125 (46.3%)	84 (42.0%)	25 (49.0%)	234 (44.9%)	82 (44.6%)	64 (38.1%)	19 (22.4%)	165 (37.8%)
Fitness center	41 (15.2%)	20 (10.0%)	3 (5.9%)	64 (12.3%)	26 (14.1%)	22 (13.1%)	15 (17.6%)	63 (14.4%)
Home	76 (28.1%)	55 (27.5%)	7 (13.7%)	138 (26.5%)	52 (28.3%)	56 (33.3%)	41 (48.2%)	149 (34.1%)
Multiple locations	79 (29.3%)	47 (23.5%)	11 (21.6%)	137 (35.7%)	59 (32.0%)	41 (24.2%)	20 (23.8%)	120 (27.6%)
Missing	1	0	0	1	0	2	1	3
**Years experience,** n(%)								
0 years	18 (6.9%)	8 (4.2%)	2 (4.1%)	28 (5.6%)	11 (6.2%)	10 (6.5%)	1 (1.2%)	22 (5.3%)
1 year	55 (21.1%)	40 (20.8%)	13 (26.5%)	108 (21.5%)	33 (18.6%)	39 (25.2%)	7 (8.5%)	79 (19.1%)
2 years	37 (14.2%)	15 (7.8%)	5 (10.2%)	57 (11.4%)	26 (14.7%)	14 (9.0%)	3 (3.7%)	43 (10.4%)
3 years	44 (16.9%)	30 (15.6%)	6 (12.2%)	80 (15.9%)	19 (10.7%)	15 (9.7%)	1 (1.2%)	35 (8.5%)
4 years	31 (11.9%)	26 (13.5%)	5 (10.2%)	62 (12.4%)	16 (9.0%)	15 (9.7%)	2 (2.4%)	33 (8.0%)
5 years	28 (10.7%)	20 (10.4%)	2 (4.1%)	50 (10.0%)	17 (9.6%)	11 (7.1%)	5 (6.1%)	33 (8.0%)
>5 years	48 (18.4%)	53 (27.6%)	16 (32.7%)	117 (23.3%)	55 (31.1%)	51 (32.9%)	63 (76.8%)	169 (40.8%)
missing	9	8	2	19	7	13	3	23
**Physical activity at least 1 day/week, n(%)**								
Crossfit	115 (42.6%)	91 (45.5%)	19 (37.3%)	225 (43.2%)	75 (40.8%)	64 (38.1%)	14 (16.5%)	153 (35.0%)
Cardio/running/swimming	108 (40.0%)	78 (39.0%)	23 (45.1%)	209 (40.1%)	77 (41.8%)	73 (43.5%)	34 (40.0%)	184 (42.1%)
Ball sports	18 (6.7%)	8 (4.0%)	1 (2.0%)	27 (5.2%)	22 (12.0%)	14 (8.3%)	8 (9.4%)	44 (10.1%)
Brisk walking	103 (38.1%)	81 (40.5%)	24 (47.1%)	208 (39.9%)	54 (29.3%)	49 (29.2%)	39 (45.9%)	142 (32.5%)
Yoga/Mobility	71 (26.3%)	53 (26.5%)	9 (17.6%)	133 (25.5%)	22 (12.0%)	27 (16.1%)	9 (10.6%)	58 (13.3%)

% can add up to more than 100%, when multiple choices were possible

#### Frequency of training

The number of training days per week differed among men and women. Among the youngest women (age 35–44), the most commonly reported number of training days per week was 5 (reported by 40.0% of women in this age category), but the most common number of days dropped to 4 days/week for the middle age category (45–59) (reported by 38.5% of women) and to 3 days/week for the oldest (60+) age category (reported by 43.1%). Among the men 4 days/week was the most common practice for both the youngest and middle age categories (35.9%), but 3 days/week was most commonly reported for ages 60 and over (45.6%). For experienced men with more than 5 years competition experience, we found that 56.2% aged 60 years train 3 days per week compared to 36.4% for less-experienced participants in the same age category. Men with at least 20 years of experience in the age group 45–59 train fewer days compared to men with less experience of the same age (p = 0.020), most commonly 3 days per week (43.5%) while the mode is 4 days for men with less experience (41.1%). They also design their own training program more frequently, 78.3% and 22.5%, respectively (p<0.001), while this is less common for women in this age group overall (10.0%).

#### Training duration

For the estimated number of training hours per week, based on the responses to “duration” and “frequency,” the great bulk of responses indicated a total weekly training time in the 5–10 hour range, for both men and women, in all age categories. Nonetheless, a decrease in total weekly training hours was evident with increasing age. A peak of about 7 hours/week was associated with the youngest men, ages 35 to 44, while most men age 75+ indicated a training time of 5 hours/week or less. Similarly, from a peak of about 8 hours/week for the youngest women, ages 35 to 44, most women aged 65+ indicated 6 hours/week or less.

#### Training location

Most women across all age categories reported training at either a weightlifting gym. There were significant age differences among the men, with most younger men (age 35–44) training at a weightlifting gym (50.5%) and only 28.3% at home, while nearly half (48.2%) of the oldest men (60+) trained at home, compared to 38.8% at a weightlifting gym. Overall 287 (29.9%) of the participants trained at home (Table 2).

#### Participation in other sports

During a given week weightlifters engaged in sports other than weightlifting such as CrossFit (43.2% of the women and 35.0% of the men), cardio, such as running, swimming, or cycling (40.1% and 42.1%) brisk walking (39.5% and 32.5%), ball sports (5.2% and 10.1%), and yoga and mobility (25.5% and 13.3%). The proportion of participants engaged in brisk walking at least one day/week increased with age and is the most common activity outside of weightlifting for lifters aged over 60 (47.1% and 45.9% for women and men, respectively).

#### Competition history

**Fig 2:**
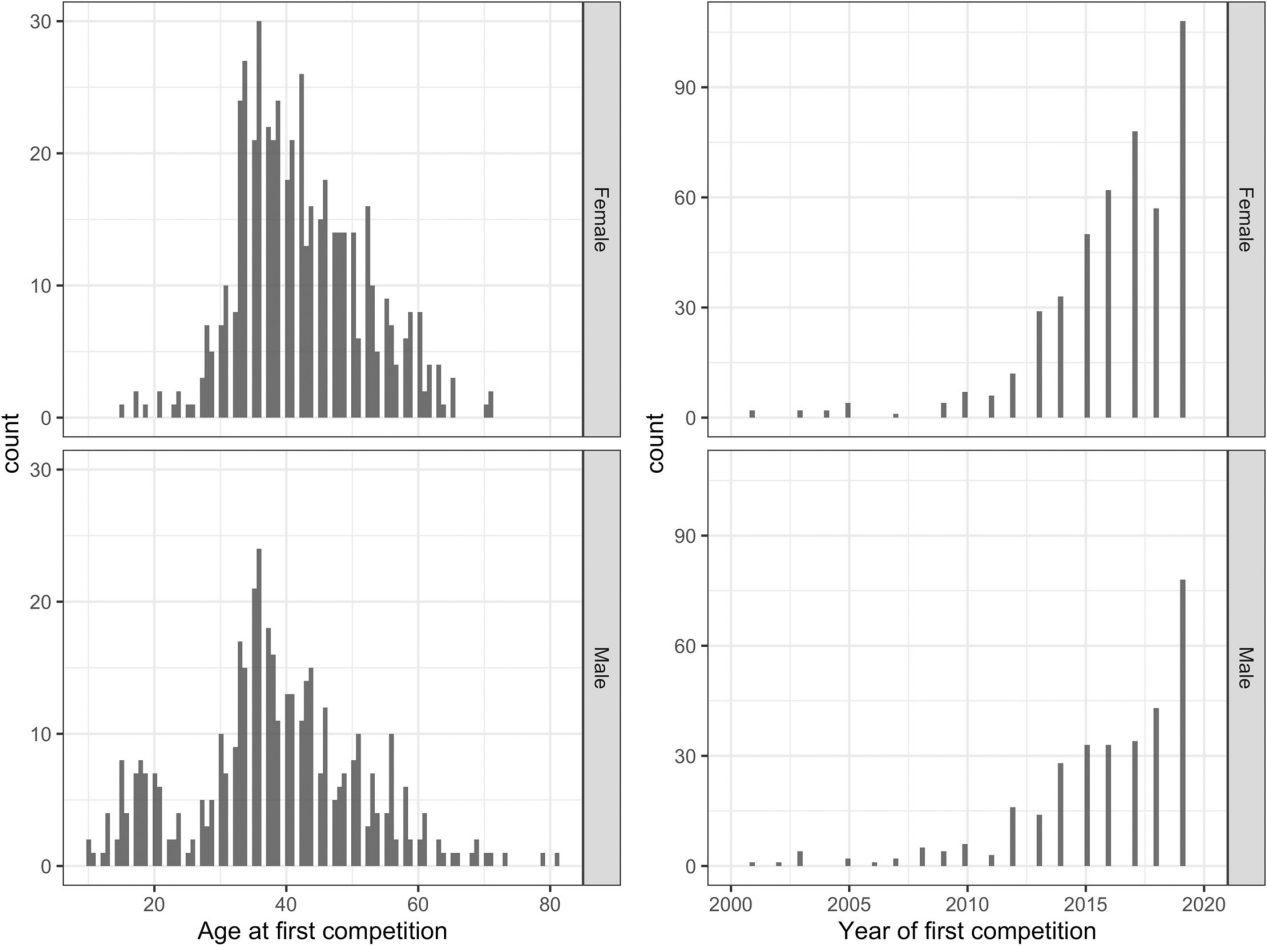
Age and year of first weightlifting competition.

Almost all surveyed participants competed in weightlifting. Only 19 (3.7%) female and 22 (5.1%) male participants did not currently compete. Over one third (35.2%) of the participants had competed at USA National Masters Championships or internationally. Male respondents have competed in weightlifting longer than women with a median (1^st^, 3^rd^ quartile) of 4 (2, 10) years for men and 3 (1, 5) years for women (p<0.001). A larger proportion of the men surveyed (40.8%) had more than 5 years of competition experience than the women (23.3%), and this was true in each of the age categories. The distribution of the age at first competition was bimodal for men with modes at ages 18 and 36, unimodal for woman with mode at age 36. Overall, 542 (59.6%) of the respondents had competed for less than 5 years (Fig 2).

Most of the participants had competition experience with sports other than weightlifting; 66.7% of women and 49.5% of men reported competing “occasionally” or “often” in CrossFit; the numbers for “Cardio” (including running, swimming, and cycling) were 61.3% and 46.5% for women and men, respectively. “Ball sports” were mentioned by 37.0% of women and 60.6% of the men, with a several other sports being mentioned by smaller numbers (S1 Table).

### Physical health and injuries

**Fig 3:**
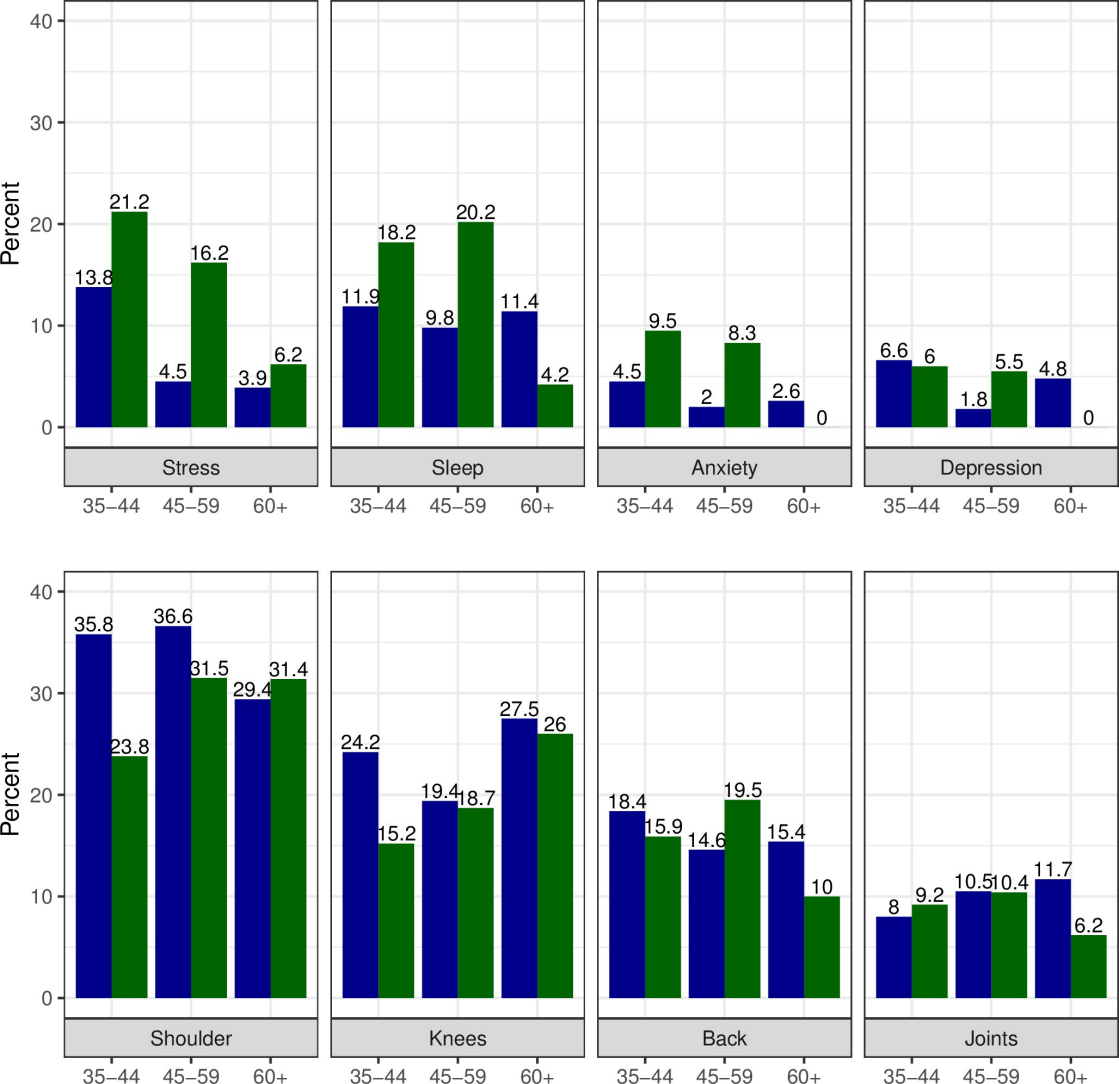
Physical and psychological factors affecting training (moderately/considerably) in the past two years for women (green) and men (blue) by age group.

The physical issues reported affecting training “moderately” or “considerably” varied, with “Shoulder” being the most common (27.9%, with little difference between men and women), followed by “Knees” (19.4%); among women; “Back,” “Hips,” and “Knees” each had similar numbers of reports (Table 3). A more detailed investigation of the shoulder issues was carried out due to their relative prevalence (Fig 3).

**Table 3:** Physical, psychological, and other factors affecting training moderately or considerably in the past two years for women and men by age group.

	Women				Men			
	Age 35–44	Age 45–59	Age 60+	Total	Age 35–44	Age 45–59	Age 60+	Total
	N = 268	N = 199	N = 51	N = 518	N = 181	N = 164	N = 84	N = 429
**Physical**								
Shoulder	63 (23.8%)	62 (31.5%)	16 (31.4%)	141 (27.5%)	36 (20.2%)	57 (35.8%)	30 (36.6%)	123 (29.4%)
Back	42 (15.9%)	38 (19.5%)	5 (10.0%)	85 (16.7%)	33 (18.4%)	22 (14.6%)	12 (15.4%)	67 (16.4%)
Hips	32 (12.2%)	30 (15.6%)	4 (8.3%)	66 (13.1%)	20 (11.4%)	12 (7.8%)	12 (15.6%)	44 (10.8%)
Knees	40 (15.2%)	36 (18.7%)	13 (26.0%)	89 (17.6%)	43 (24.2%)	30 (19.4%)	22 (27.5%)	95 (23.0%)
Joints	24 (9.2%)	20 (10.4%)	3 (6.2%)	47 (9.4%)	14 (8.0%)	16 (10.5%)	9 (11.7%)	39 (9.6%)
**Psychological**								
Anxiety	25 (9.5%)	16 (8.3%)	0 (0.0%)	41 (8.2%)	8 (4.5%)	3 (2.0%)	2 (2.6%)	13 (3.2%)
Depression	16 (6.0%)	11 (5.5%)	0 (0.0%)	27 (5.2%)	12 (6.6%)	3 (1.8%)	4 (4.8%)	19 (4.4%)
Stress	56 (21.2%)	31 (16.2%)	3 (6.2%)	90 (17.9%)	24 (13.8%)	7 (4.5%)	3 (3.9%)	34 (8.4%)
Sleep	8 (18.3%)	39 (20.2%)	2 (4.2%)	89 (17.7%)	21 (11.9%)	15 (9.8%)	9 (11.4%)	45 (11.0%)
**Health**								
Cardiovascular	1 (0.4%)	0 (0.0%)	0 (0.0%)	1 (0.2%)	3 (1.7%)	2 (1.3%)	5 (6.4%)	10 (2.5%)
Cancer	1 (0.4%)	5 (2.6%)	1 (2.1%)	7 (1.4%)	1 (0.6%)	2 (1.3%)	4 (5.1%)	7 (1.7%)
Diabetes	0 (0.0%)	0 (0.0%)	0 (0.0%)	0 (0.0%)	0 (0.0%)	0 (0.0%)	2 (2.6%)	2 (0.5%)
Autoimmune diseases	3 (1.2%)	5 (2.6%)	1 (2.1%)	9 (1.8%)	0 (0.0%)	4 (2.6%)	2 (2.5%)	6 (1.5%)
Respiratory Diseases	5 (1.9%)	2 (1.1%)	0 (0.0%)	7 (1.4%)	1 (0.6%)	3 (2.0%)	0 (0.0%)	4 (1.0%)
Digestive diseases	1 (0.4%)	3 (1.6%)	1 (2.1%)	5 (1.0%)	1 (0.6%)	0 (0.0%)	0 (0.0%)	1 (0.2%)
Chronic diseases	4 (1.5%)	2 (1.1%)	1 (2.1%)	7 (1.4%)	0 (0.0%)	0 (0.0%)	6 (7.7%)	6 (1.5%)
**Life circumstances**								
Work demands	162 (60.4%)	91 (45.7%)	18 (35.3%)	271 (52.3%)	67 (37.0%)	67 (40.9%)	55 (65.5%)	189 (44.1%)
Access to facility	22 (8.2%)	10 (5.0%)	4 (7.8%)	36 (6.9%)	22 (12.2%)	12 (7.3%)	5 (6.0%)	39 (9.1%)
Financial constraints	13 (4.9%)	7 (3.5%)	1 (2.0%)	21 (4.1%)	6 (3.3%)	4 (2.4%)	1 (1.2%)	11 (2.6%)
Child care	38 (14.2%)	9 (4.5%)	0 (0.0%)	47 (9.1%)	36 (19.9%)	11 (6.7%)	3 (3.6%)	50 (11.7%)
Elder care	3 (1.1%)	7 (3.5%)	2 (3.9%)	12 (2.3%)	3 (1.7%)	2 (1.2%)	1 (1.2%)	6 (1.4%)
Family concerns	36 (13.4%)	24 (12.1%)	2 (3.9%)	62 (12.0%)	24 (13.3%)	15 (9.1%)	3 (3.6%)	42 (9.8%)
**Pregnancy**	10 (3.7%)	1 (0.5%)	0 (0.0%)	11 (2.1%)				
**Menstruation**	18 (6.9%)	2 (1.1%)	0 (0.0%)	20 (4.0%)				
**Menopausal Symptoms**	6 (2.3%)	25 (12.8%)	0 (0.0%)	31 (6.1%)				

**Fig 4:**
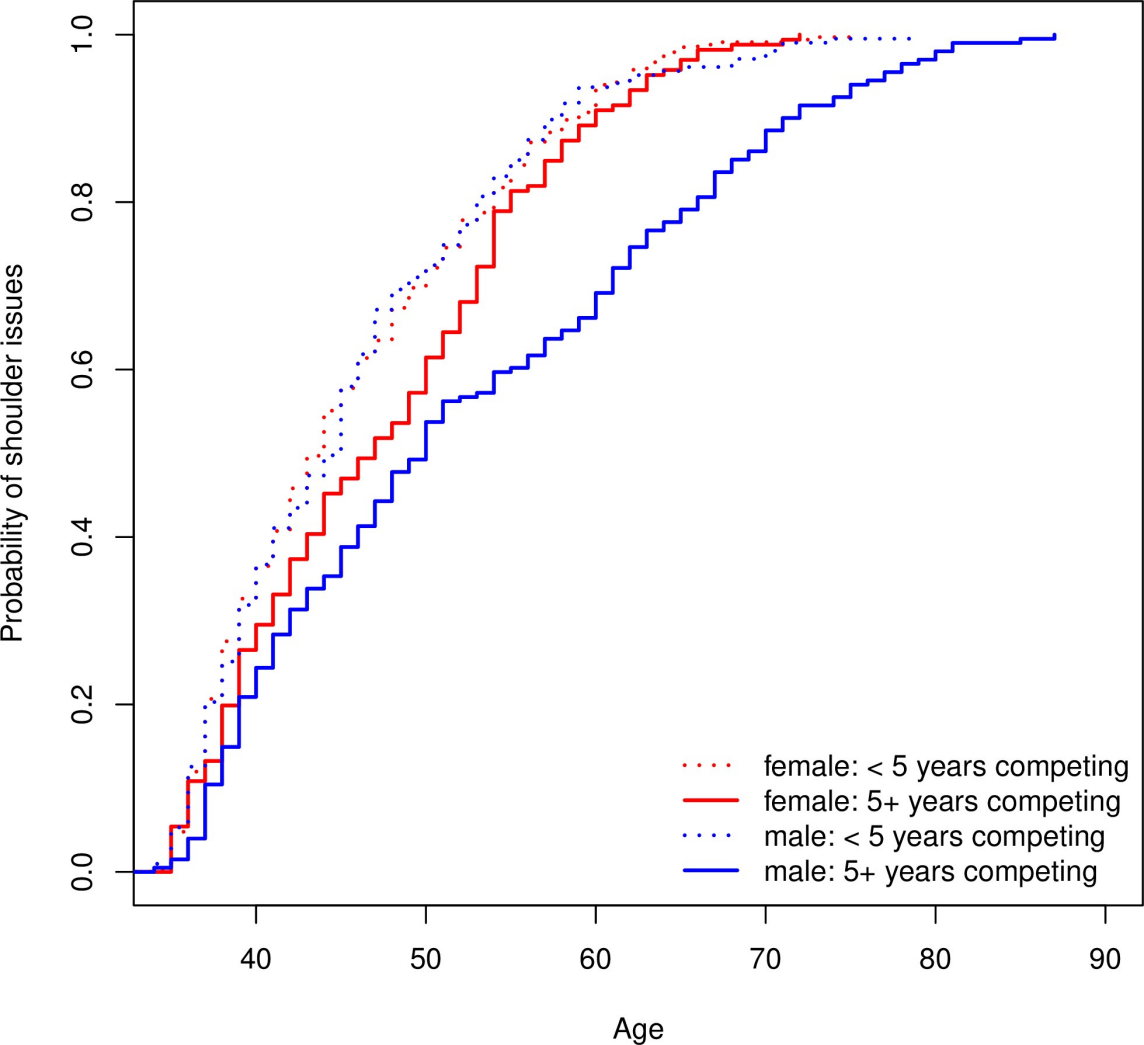
Cumulative incidence curve for age at which shoulder problems affected training stratified by participants who had 5 years or more experience in competitions versus those with less.

We found that the probability of having suffered shoulder issues increased with age for both men and women, as expected. Between men and women with less than 5 years of competition experience, there was almost no difference in the injury pattern as a function of age. However, among those with more than 5 years of competition experience, probability of shoulder injury was significantly greater for the women at every age, with the disparity widening considerably after age 50–55 (p<0.001) (Fig 4).

Lengthy training interruptions (more than 1 month) were reported by significant numbers of both women and men, with “Sport injury” being the most common cause, cited by 29.9% of women and 26.6% of men. The next most common cause was “Work demands,” reported by 15.8% of women and 17.5% of men (S2 Table).

Health challenges such as cancer (1.5%) or chronic diseases (1.4%) affect training moderately or considerably, but are relatively rare among this group. In the age group older than 60 years, men are affected more than women by cardiovascular disease (6.4% vs 0%) and chronic disease (7.7% vs 2.2%).

### Psychological and lifestyle factors

Stress levels constrain/restrict training more for women than for men with 17.9% and 8.4%, respectively (p<0.001). Disruptions due to “Sleep” were reported by 17.7% and 11.0%, for women and men, respectively, (p = 0.014). (Table 3, Fig 3)

Depression or anxiety was more common in women than in men with 9.8% and 5.4%, respectively, (p = 0.024).

“Work demands” were cited by 52.3% of women and 44.1% of men as having moderate or considerable effect on training. There was a higher prevalence in younger women, 35–44 years, versus older women, 60+ years, (60.4% and 35.3%, respectively). This was reversed for younger versus older men (37.0% and 65.5%, respectively). There was no significant difference in the reporting of family concerns, including child care and elder care, with 18.9% of women and 17.2% of men (p = 0.639) reporting such concerns.

### Women and menopause

#### Pregnancy

Thirteen women reported factors associated with pregnancy. Nine women mentioned taking time off for pregnancy, typically between 1 and 6 months (n = 6), others more than one year (n = 2).

#### Menopause

**Fig 5:**
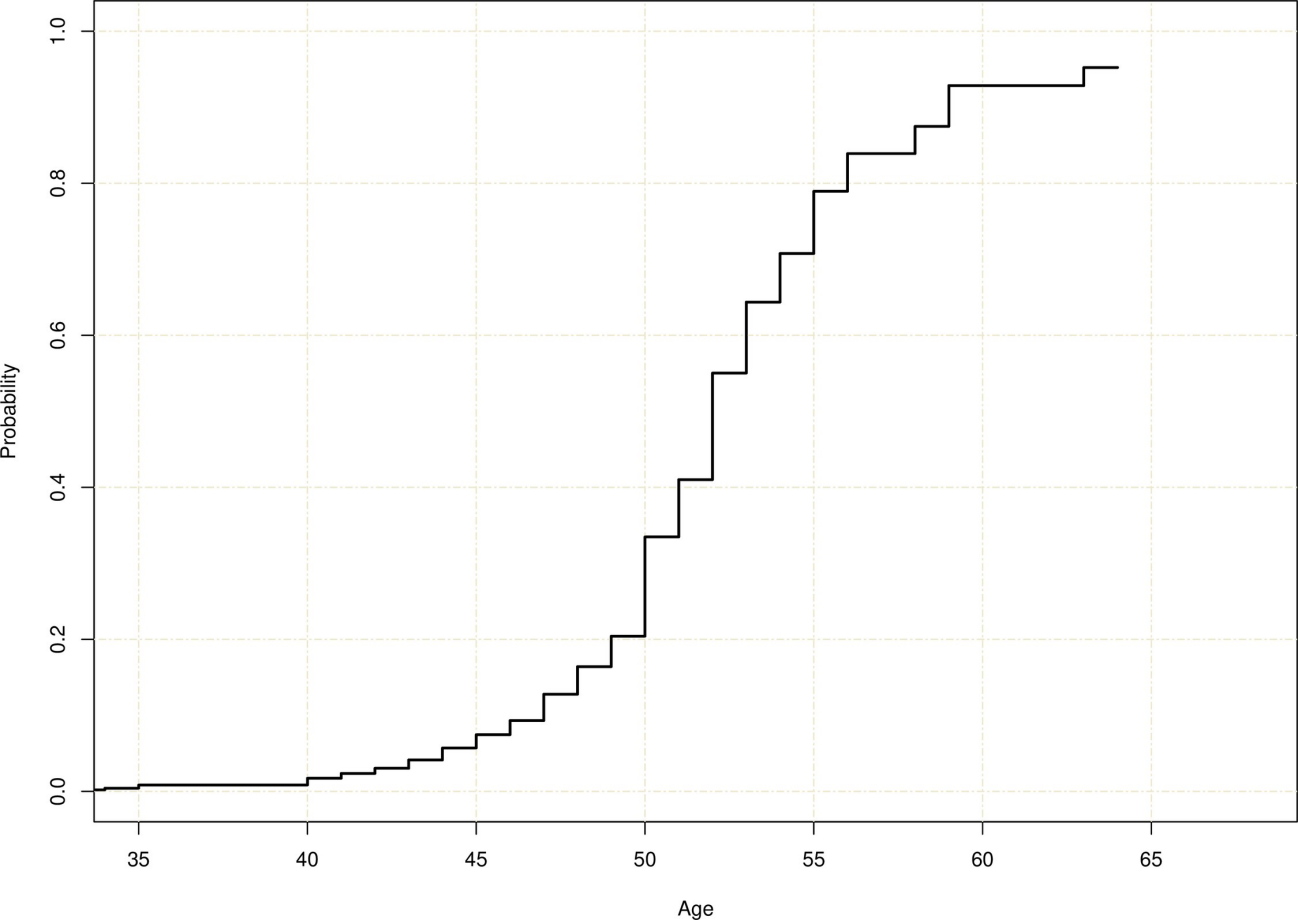
Cumulative incidence curve for the natural age of menopause (based on n = 481 women of whom 133 had reached natural menopause, defined by 1 year of no menses).

Menopause was defined in the survey as not having menstruated for one year. Among 132 menopausal women peri/menopausal symptoms affected training in 31 women ‘moderately’ or ‘considerably.’ Most commonly women had trouble sleeping (43.2%) and experienced fatigue (33.8%), weight gain (28.1%), hot flashes (24.4%), or mood swings (10.0%), while 34.5% did not report symptoms. There were 18 (13.6%) who achieved natural menopause before age 45, 97 (73.5%) between 45 and 54, and 17 (12.9%) at 55 or older. A 95% confidence interval for the median age at menopause is (50, 52) years. The cumulative incidence function representing the rate of menopause at a given age is shown in Fig 5. Histograms of reported age at menopause and ages of women who had not experienced menopause are shown in S2 Fig. In addition, there were 6 women who had medically or surgically induced menopause, 7 menopausal women had missing age at menopause, 1 identified as transgender.

## Discussion

Many health benefits are attributed to vigorous physical activity and resistance training, such as for cardiovascular and bone health, mobility, improved function in daily living activities, and maintaining an independent lifestyle [22, 23]. Thus it is of interest to examine the physical activities and health characteristics of older individuals who are dedicated to sports training or competition, such as Masters athletes. This is the first study to examine training habits and health challenges in a large population of Masters weightlifters ages 34 to 87, and one of the few to examine health and activity characteristics of older individuals involved in competitive sport.

It should be emphasized that there have occurred vast and rapid changes in the sport of weightlifting during the past 10 years, where the CrossFit phenomenon has introduced many thousands of individuals to the once-niche sport of Olympic-style weightlifting. Thus, the population surveyed is not, for the most part, composed of individuals who have participated in weightlifting training or competition since their youth; instead, they are predominantly novices in the sport with less than 5 years of experience. Among the women in particular, more than three-quarters had started within the past 5 years, and only one in twenty had started before the age of 30. In comparison, a Canadian study of track and field Masters athletes in 2015 reported that only 28% had competed less than 5 years [24]. However, these differences in previous sports experience may not ultimately have much impact, as it has been shown for runners that both early-starting Masters athletes (such as those who trained for most of their adulthood) and late-starting Masters (such as those who started training at age 50), can both achieve the same level of performance [25].

Physical, psychological and social benefits for competitive athletes in a variety of sports have been investigated in older adults [22] and perceptions of such benefits have been examined for participants at World Masters Games [26, 27]. While prior studies have focused on older athletes competing at the world championships level (6,8,11), it is equally important to understand the benefits and obstacles of Masters sports at a broader, mass-participation level. In this study we were able to examine the demographic, training, and health-related details of a cohort of older adults who engage in one particular sport at a range of levels, from elite world champions to those who participate only at local clubs and recreation centers. The sport of weightlifting has been growing in recent years with participants at all ages, many without prior experience in weightlifting, and some without being physically active for many years. In this sense, the rapidly expanding Masters weightlifting phenomenon can be seen as a challenge to negative stereotypes of aging that include increasing affliction by disease and poor quality of life [26, 28]. Surveyed participants are motivated to stay engaged by a strong desire to train; they enjoy the social interactions, and their increased strength that they can apply to more safely perform daily living activities. Obstacles encountered by the participants include injuries or other health issues, lack of available time, family concerns, or insufficient financial resources. Lack of access to facilities, and environments that focus on younger athletes were also mentioned.

This is a highly educated and affluent group;85% were post-secondary graduates. The great majority were white (84%), and most were married (72%). It is important to note that similar distributions have also been observed in other studies of Masters athletes. For example, study of World Masters Games athletes in 2001 found that 55% were post-secondary graduates [29], while 90% were white and 70% were married, while a Canadian study of track and field Masters athletes in 2015 reported 48% married and 70% post-secondary graduates [24]. More research on social and racial disparities in sports [30] and Masters sports is needed.

### Weightlifting training habits

Over half of the survey participants are concurrently engaged in other sports and three quarters had competition experience prior to weightlifting. In comparison, over half of the 2001 World Masters Games athletes participated in multi-sports [29]. The majority of the weightlifters had participated in a local weightlifting competition, and more than half competed at higher levels in the USA National Masters or in international weightlifting competitions. Thus, the participants not only engage in exercise, they also challenge themselves and set goals for competitions.

Masters weightlifters across all age groups train “long and often” (1–2 hours, 3–5 days/week) and also engage in multiple sports. This surpasses the exercise guidelines from the U.S. Center for Disease Control and Prevention and the American College of Sports Medicine of 3–5 days per week for 20–60 minute sessions with 2–3 days per week resistance training [6]. For older athletes an overall reduction in training “stimulus” has been observed [13], referring to exercise-training intensity, session duration, and weekly frequency. Naturally, there is a decline in the actual weights lifted as athletes age. Nevertheless, in this cohort of weightlifters, the length of a training session was 1 hour or more for over 86% of the women across all age groups. For men, this proportion declined from 90% (in the youngest age category) to 76% (in the oldest age category). Three days per week was the most commonly reported training frequency for the oldest age group at 60 and over; while four- to five-days per week training was common at younger ages. Men with at least 20 years of experiences in the age group 45–59, train fewer days and in shorter sessions compared to men with less experience of the same age; they also design their own training program more frequently. We have no data regarding “intensity” of training defined as percentage of maximum weights lifted, although anecdotes suggest that this generally declines with age as a safeguard against injury. In comparison, a study on Masters runners ages 40 and up reported that healthier runners have fewer running sessions per week, which also had the effect of decreasing the rate of injury [31]. The reality of having to adjust training regimes to accommodate aging bodies needs to be acknowledged by athletes and coaches in order to train more safely and not run the risk of overtraining that might lead to injuries. While the peak starting age for weightlifting for most survey participants was 34 or 35, for both women and men, a subgroup of 15% of the men had started training as teenagers or in their early twenties. In some sports, a greater proportion of males participate at a younger age than females. The reasons are not known, but boys may be encouraged more to participate in sport at a young age than girls [32]. However, weightlifting for women is a relatively new phenomenon, with Olympic women’s competition not having started until the year 2000. By contrast, women have competed in Olympic track and field athletics since 1928, and in Olympic swimming since 1912.

### Physical health and injuries

It is notable that only a very small proportion of Masters weightlifters reported that chronic diseases had affected training moderately or considerably, although it is higher in those ages 60 and over. This could be due to the fact that sport participants have a significantly lower prevalence of chronic diseases compared to non-athletes [24, 33]. It is possible that only the healthiest athletes continue to train at older ages. On the other hand, while chronic diseases and injuries affect their training, as reported in this study, it is of note that Masters athletes continue to train through setbacks and obstacles [28]. Engagement in athletic activities carries an increased injury risk [24, 33] which results in time loss from training or competition even if injuries are not severe. Injury rates in athletes and active older adults have been studied in different populations [17, 31, 34–36]. We found that over a 2-year period, shoulder issues affected training moderately or considerably for 28% of Masters weightlifters, while knee and back problems had a similar effect for 23% and 16%, of weightlifters respectively, with little difference between men and women. Previous studies on injury rates in Olympic weightlifting have focused on younger athletes. For elite weightlifters ages 18 to 37 there were 2.4 to 3.3 injuries per 1000 hours of training [37–39] and shoulder injuries were the most commonly reported affliction, at a rate of 0.42 per 1000 hours [38]. For junior weightlifting athletes at the US Olympic Training Center, the most common afflictions were knee and shoulder injuries with 19% and 18% affected, respectively, although 90% missed training time of less than one week [39]. In contrast, older athletes in a variety of sports at the 2001 World Masters Games reported most commonly lower back pain (25%) and knee osteoarthritis (15%) [29], and Masters runners at an international competition reported an injury rate of 49% in the previous year with knee injuries accounting for 19% [31]. Over a 12-month period, 70% of male track and field Masters athletes reported an injury compared to 15% of moderately active older adults [24]. Not surprisingly, the specific type of sport can be associated with the anatomical location of an overuse injury; for Olympic weightlifters, knee and shoulders are commonly reported locations for chronic injuries, while acute injuries are often related to tendon ruptures, especially at older ages [40]. Many survey participants engage at least one day each week in other sports such as running, cycling, and swimming, CrossFit, or ball sports. Several commented that their injuries resulted from other sports or from prior injuries. This is congruent with a Finnish study of top-level competitive athletes aged 15–35, in which it was found that the majority of the many injuries occurred in sports other than the main one [41]. Also, competitive athletes reported that for 36 to 38% of acute or overuse injuries there was a prior injury at the same location [41]. In comparison, injury rates in a Finnish active-living study were highest in ball sports, contact, and team sports, with 6.6 to 18.3 per 1000 hours [36]. The reported rates of injury-imposed restrictions on weightlifting training over a 2-year period in this population are lower than reported injury rates in running or other sports over a 1-year period. However, the prevalence of injuries could be higher in this population, since injuries were only reported if they restricted training moderately or considerably. Taking into account that a large proportion of these injuries may be due to other activities or prior injuries, the findings suggest that it is important for older adults, their coaches, and health providers to acknowledge the contribution of type of physical activities, training load, and weightlifting technique on safety, especially when coupled with underlying medical conditions such as old injuries.

Research studies on gender differences in sport injuries are limited [14, 42, 43]. This could be due to the sport discipline studied, a focus on younger athletes, or the type of study (intervention versus retrospective observational study). An important finding of this study was the age and gender disparity in shoulder problems mediated by years of competition experience. There is a difference between weightlifters who are relatively new to the sport with less than 5 years competition experience compared to those who trained in weightlifting longer. Those with less experience have a higher probability of shoulder issues with no difference between men and women. Among weightlifters with competition experience of 5 years or more, younger female Masters athletes have the same probability of shoulder issues as younger males, but there is a higher probability of shoulder injuries for women after age 50 when compared with men of the same age. This is congruent to a prior study of an urban population across all ages where more shoulder injuries were observed in older women compared to older men in a trauma center, and there was a rise in incidence rates for women as they age [43].

### Psychological and lifestyle factors

Work demands have a moderate or considerable effect on training for 52% of the women and 44% of the men in our sample, with a higher prevalence in younger women, 35–44 years, versus older women, 60+ years. Stress levels constrain or restrict training more for women than for men with 18% and 8%, respectively. Depression and anxiety were twice as likely in female weightlifters than in males with 10% and 5%, respectively. These numbers correspond almost exactly to the numbers found in the general population, as reported by the Center for Disease and Control [15]. They are comparable to the prevalence of anxiety disorders among individuals from Euro/Anglo cultures (4–10%) which are higher than in other cultures [44, 45], but older adults report less worry than younger adults [46]. In addition to the physical benefits, the psychological benefits associated with physical activity among older adults have been well documented [5, 27, 33] we found these benefits reflected by our survey participants, many of whom commented on improved mental health when training in weightlifting.

Despite the reported improvement in sleep quality due to exercise, sleep disturbances affected 18% of women’s training “moderately” or “considerably” while this percentage was 11% for men. It is known that sleep disturbances are common in older women, affecting more than 40% of perimenopausal and postmenopausal women [47, 48], congruent with our study where 43% of postmenopausal women reported having trouble sleeping. In turn, poor sleep has been shown to be associated with poorer physical performance in other studies [49, 50], an observation that has also been made by participants in our study.

### Menopause

Physical activity is associated with changes in hormonal levels. Demographic, genetic, and lifestyle factors may affect the age at menopause [51]. We hypothesized that menopause among women weightlifters may occur later than in the general population. However, the median age at menopause was 50 years among this weightlifting population, which corresponds to the median age among white women from industrialized countries. It is of note that 13% achieved natural menopause after age 55. Menopausal symptoms, such as trouble sleeping, fatigue, weight gain, or hot flashes, had affected training in 23% of the postmenopausal women. It is important to be aware of such age profiles to inform women and health care providers, to take into account for treatment decisions regarding presentations of menopause. This may affect training choices since some hormonal therapies are prohibited under the World Anti-Doping Agency (WADA) for all competitive athletes (https://www.wada-ama.org/en/content/what-is-prohibited).

#### Limitations

This study is based on voluntary participation of individuals. This may have an effect on the observed risk factor distribution and on self-reported disease. We were not able to verify self-reported diagnoses. The study population was comprised of highly educated and predominantly white older adults. It would be important to study underrepresented groups among the Masters athletes. However, the findings allow for insights regarding athletic performance potential and challenges for highly active older adults, as well as the health profiles of Masters weightlifters in particular. The cross-sectional design gives rise to a potential bias at the older age groups with the possibility of a relatively healthier population than non-athletes in their age cohort, compared to the younger ages in which the health differences between athletes and non-athletes may be much smaller. While there has been a dramatic increase in the participation of women, they are still underrepresented at ages older than 65.

## Conclusion

Older athletes are capable of rigorous training programs and top performances while adjusting to changes due to biological aging. To our knowledge this is the first study to comprehensively describe the demographics, training habits, and lifestyle factors of a cohort of Masters weightlifters ranging from beginners at different ages to top level or experienced athletes. We are able to observe health conditions and injuries disrupting training and discuss gender differences. Weightlifting athletes, coaches, and health professionals must be aware of patterns of injuries and gender differences to incorporate successful prevention strategies. The age profile at onset of menopause and the impact of menopausal symptoms on weightlifting training provides new information.

## Supporting information

S1 Table Competition experience with sports other than weightlifting. (DOCX)

S2 Table Training interruptions in the past two years for more than 1 month. (DOCX)

S1 Fig STROBE flow diagram. (TIF)

S2 Fig Histogram of age at menopause and ages for women without menopause. (TIFF)

S1 File (PDF)
