# Correction: The Masters athlete in Olympic weightlifting: Training, lifestyle, health challenges, and gender differences

**DOI:** 10.1371/journal.pone.0247110

**Published:** 2021-02-10

**Authors:** Marianne Huebner, David E. Meltzer, Wenjuan Ma, Holly Arrow

The second author’s name is spelled incorrectly. The correct name is: David E. Meltzer. The correct citation is: Huebner M, Meltzer DE, Ma W, Arrow H (2020) The Masters athlete in Olympic weightlifting: Training, lifestyle, health challenges, and gender differences. PLoS ONE 15(12): e0243652. https://doi.org/10.1371/journal.pone.0243652
