# Correction: The Masters athlete in Olympic weightlifting: Training, lifestyle, health challenges, and gender differences

**DOI:** 10.1371/journal.pone.0305284

**Published:** 2024-06-06

**Authors:** Marianne Huebner, David Meltzer, Wenjuan Ma, Holly Arrow

There is an error in Table 1. The values in the category “Income” are incomplete. Please see the correct Table 1 below.

**Table 1:** Demographics by gender.

	Women (n = 521)	Men (n = 437)	Total (n = 958)
**Age**, median (range)	44 (34–75)	46 (34–87)	45 (34–87)
**Age groups,** n(%)			
35–39	159 (30.5%)	116 (26.5%)	275 (28.7%)
40–44	111 (21.3%)	68 (15.6%)	179 (18.7%)
45–49	74 (14.2%)	79 (18.1%)	153 (16.0%)
50–54	77 (14.8%)	50 (11.4%)	127 (13.3%)
55–59	49 (9.4%)	39 (8.9%)	88 (9.2%)
60–64	35 (6.7%)	29 (6.6%)	64 (6.7%)
65–69	11 (2.1%)	22 (5.0%)	33 (3.4%)
70–74	4 (0.8%)	18 (4.1%)	22 (2.3%)
75–79	1 (0.2%)	10 (2.3%)	11 (1.1%)
80+	0 (0.0%)	6 (1.4%)	6 (0.6%)
**Marital Status,** n(%)			
Married	339 (65.6%)	343 (78.7%)	682 (71.6%)
Divorced/widowed/separated	74 (14.4%)	35 (8.0%)	109 (11.4%)
Not married	104 (20.1%)	58 (13.3%)	162 (17.0%)
missing	4	1	5
**Race,** n(%)			
White/Caucasian	425 (82.5%)	369 (85.6%)	794 (83.9%)
Black or African American	19 (3.7%)	10 (2.3%)	29 (3.1%)
Asian	25 (4.9%)	20 (4.6%)	45 (4.8%)
Native Hawaiian or Pacific Islander	6 (1.2%)	5 (1.2%)	11 (1.2%)
American Indian or Alaska Native	1 (0.2%)	1 (0.2%)	2 (0.2%)
Multiple + other	39 (7.5%)	26 (5.9%)	65 (6.8%)
Missing/Decline to answer	6	6	12
**Hispanic**, yes (%)	46 (8.9%)	34 (7.8%)	80 (8.4%)
Missing/Decline to answer	3	2	5
**Income,** n(%)			
<$100,000	165 (33.7%)	131 (31.4%)	296 (32.7%)
$100,000-$149,000	110 (22.5%)	110 (26.3%)	220 (24.3%)
$150,000 - $200,000	73 (14.9%)	71 (17.0%)	144 (15.9%)
>$200,000	99 (20.2%)	87 (20.8%)	186 (20.5%)
Decline to Answer	42 (8.6%)	19 (4.5%)	61 (6.7%)
Missing	32	19	51
**Employment,** n(%)			
Full-time	409 (78.7%)	359 (82.5%)	768 (80.4%)
Part-time	67 (12.9%)	26 (6.0%)	93 (9.7%)
Retired	28 (5.4%)	41 (9.4%)	69 (7.2%)
Unemployed	16 (3.1%)	9 (2.1%)	25 (2.6%)
Missing/Decline to answer	1	2	3
**Education,** n(%)			
High school/GED or less	14 (2.6%)	17 (3.9%)	31 (3.2%)
Some college	64 (12.3%)	79 (18.1%)	143 (14.9%)
College degree	165 (31.7%)	162 (37.1%)	327 (34.1%)
Some graduate school	35 (6.7%)	20 (4.6%)	55 (5.7%)
Graduate school	243 (46.6%)	159 (36.4%)	402 (42.0%)
