# Supplementary material for: The Masters athlete in Olympic weightlifting: Training, lifestyle, health challenges, and gender differences
Source: PLoS One. 2020 Dec 4;15(12):e0243652. doi: 10.1371/journal.pone.0243652 (PMC7717526; doi:10.1371/journal.pone.0243652)
Supplement: S1 Table — (DOCX) [file pone.0243652.s001.docx]

**S1 Table. Competition experience with sports other than weightlifting**

|  | **Women** | | | | **Men** | | | |
| --- | --- | --- | --- | --- | --- | --- | --- | --- |
|  | **Age 35-44** | **Age 45-59** | **Age 60+** | **Total** | **Age 35-44** | **Age 45-59** | **Age 60+** | **Total** |
|  | N=270 | N=200 | N=51 | N=521 | N=184 | N=168 | N=85 | N=437 |
| **Crossfit** |  |  |  |  |  |  |  |  |
| Never | 78 (29.0%) | 72 (36.0%) | 23 (45.1%) | 173 (33.3%) | 69 (37.7%) | 82 (49.1%) | 69 (81.2%) | 220 (50.6%) |
| Occasionally | 113 (42.0%) | 90 (45.0%) | 18 (35.3%) | 221 (42.5%) | 67 (36.6%) | 52 (31.1%) | 7 (8.2%) | 126 (29.0%) |
| Often | 78 (29.0%) | 38 (19.0%) | 10 (19.6%) | 126 (24.2%) | 47 (25.7%) | 33 (19.8%) | 9 (10.6%) | 89 (20.5%) |
| Missing | 1 | 0 | 0 | 1 | 1 | 1 | 0 | 2 |
| **Cardio (running, swimming, cycling)** |  |  |  |  |  |  |  |  |
| Never | 100 (37.0%) | 73 (36.7%) | 28 (54.9%) | 201 (38.7%) | 91 (49.5%) | 94 (56.0%) | 49 (57.6%) | 234 (53.5%) |
| Occasionally | 105 (38.9%) | 72 (36.2%) | 12 (23.5%) | 189 (36.3%) | 56 (30.4%) | 46 (27.4%) | 18 (21.2%) | 120 (27.5%) |
| Often | 65 (24.1%) | 54 (27.1%) | 11 (21.6%) | 130 (25.0%) | 37 (20.1%) | 28 (16.7%) | 18 (21.2%) | 83 (19.0%) |
| Missing | 0 | 1 | 0 | 1 |  |  |  |  |
| **Track and Field** |  |  |  |  |  |  |  |  |
| Never | 217 (80.4%) | 142 (71.0%) | 40 (78.4%) | 399 (76.6%) | 141 (76.6%) | 130 (77.4%) | 57 (67.1%) | 328 (75.1%) |
| Occasionally | 31 (11.5%) | 28 (14.0%) | 4 (7.8%) | 63 (12.1%) | 18 (9.8%) | 22 (13.1%) | 11 (12.9%) | 51 (11.7%) |
| Often | 22 (8.1%) | 30 (15.0%) | 7 (13.7%) | 59 (11.3%) | 25 (13.6%) | 16 (9.5%) | 17 (20.0%) | 58 (13.3%) |
| **Gymnastics, cheer leading** |  |  |  |  |  |  |  |  |
| Never | 216 (80.0%) | 166 (83.0%) | 46 (90.2%) | 428 (82.1%) | 176 (95.7%) | 162 (96.4%) | 79 (92.9%) | 417 (95.4%) |
| Occasionally | 25 (9.3%) | 16 (8.0%) | 2 (3.9%) | 43 (8.3%) | 2 (1.1%) | 3 (1.8%) | 3 (3.5%) | 8 (1.8%) |
| Often | 29 (10.7%) | 18 (9.0%) | 3 (5.9%) | 50 (9.6%) | 6 (3.3%) | 3 (1.8%) | 3 (3.5%) | 12 (2.7%) |
| **Ball sports** |  |  |  |  |  |  |  |  |
| Never | 160 (59.3%) | 129 (64.5%) | 39 (76.5%) | 328 (63.0%) | 60 (32.6%) | 72 (43.4%) | 39 (46.4%) | 171 (39.4%) |
| Occasionally | 40 (14.8%) | 28 (14.0%) | 5 (9.8%) | 73 (14.0%) | 47 (25.5%) | 40 (24.1%) | 16 (19.0%) | 103 (23.7%) |
| Often | 70 (25.9%) | 43 (21.5%) | 7 (13.7%) | 120 (23.0%) | 77 (41.8%) | 54 (32.5%) | 29 (34.5%) | 160 (36.9%) |
| Missing |  |  |  |  | 0 | 2 | 1 | 3 |
| **Power lifting** |  |  |  |  |  |  |  |  |
| Never | 229 (84.8%) | 165 (82.5%) | 44 (86.3%) | 438 (84.1%) | 148 (80.4%) | 124 (73.8%) | 51 (60.0%) | 323 (73.9%) |
| Occasionally | 34 (12.6%) | 28 (14.0%) | 6 (11.8%) | 68 (13.1%) | 26 (14.1%) | 25 (14.9%) | 26 (30.6%) | 77 (17.6%) |
| Often | 7 (2.6%) | 7 (3.5%) | 1 (2.0%) | 15 (2.9%) | 10 (5.4%) | 19 (11.3%) | 8 (9.4%) | 37 (8.5%) |
| **Martial arts, wrestling, boxing** |  |  |  |  |  |  |  |  |
| Never | 241 (89.3%) | 180 (90.0%) | 48 (94.1%) | 469 (90.0%) | 133 (72.3%) | 123 (73.2%) | 70 (82.4%) | 326 (74.6%) |
| Occasionally | 17 (6.3%) | 14 (7.0%) | 3 (5.9%) | 34 (6.5%) | 20 (10.9%) | 26 (15.5%) | 7 (8.2%) | 53 (12.1%) |
| Often | 12 (4.4%) | 6 (3.0%) | 0 (0.0%) | 18 (3.5%) | 31 (16.8%) | 19 (11.3%) | 8 (9.4%) | 58 (13.3%) |
| **Skiing, skating** |  |  |  |  |  |  |  |  |
| Never | 264 (97.8%) | 192 (96.0%) | 51 (100.0%) | 507 (97.3%) | 173 (94.0%) | 153 (91.1%) | 80 (94.1%) | 406 (92.9%) |
| Occasionally | 6 (2.2%) | 7 (3.5%) | 0 (0.0%) | 13 (2.5%) | 8 (4.3%) | 13 (7.7%) | 4 (4.7%) | 25 (5.7%) |
| Often | 0 (0.0%) | 1 (0.5%) | 0 (0.0%) | 1 (0.2%) | 3 (1.6%) | 2 (1.2%) | 1 (1.2%) | 6 (1.4%) |

Competition in other sports: Equestrian 16 (3.1%) Women only (often=11, never=2).
