# Supplementary material for: The Masters athlete in Olympic weightlifting: Training, lifestyle, health challenges, and gender differences
Source: PLoS One. 2020 Dec 4;15(12):e0243652. doi: 10.1371/journal.pone.0243652 (PMC7717526; doi:10.1371/journal.pone.0243652)
Supplement: S2 Table — (DOCX) [file pone.0243652.s002.docx]

**S2 Table. Training interruptions in the past two years for more than 1 month**

|  | **Women** | | | | **Men** | | | |
| --- | --- | --- | --- | --- | --- | --- | --- | --- |
|  | **Age 35-44** | **Age 45-59** | **Age 60+** | **Total** | **Age 35-44** | **Age 45-59** | **Age 60+** | **Total** |
|  | N=268 | N=199 | N=51 | N=518 | N=181 | N=164 | N=84 | N=429 |
| Work demands | 49 (18.3%) | 24 (12.1%) | 9 (17.6%) | 82 (15.8%) | 43 (23.8%) | 23 (14.0%) | 9 (10.7%) | 75 (17.5%) |
| Access to facility | 15 (5.6%) | 7 (3.5%) | 7 (13.7%) | 29 (5.6%) | 6 (3.3%) | 6 (3.7%) | 2 (2.4%) | 14 (3.3%) |
| Other sports | 8 (3.2%) | 10 (5.5%) | 0 (0.0%) | 18 (3.8%) | 6 (3.5%) | 2 (1.3%) | 3 (4.2%) | 11 (2.8%) |
| Sport injury | 80 (29.9%) | 65 (32.7%) | 10 (19.6%) | 155 (29.9%) | 51 (28.2%) | 41 (25.0%) | 22 (26.2%) | 114 (26.6%) |
| Injury | 18 (6.7%) | 26 (13.1%) | 2 (3.9%) | 46 (8.9%) | 13 (7.2%) | 15 (9.1%) | 11 (13.1%) | 39 (9.1%) |
| Surgery | 21 (7.8%) | 28 (14.1%) | 3 (5.9%) | 52 (10.0%) | 18 (9.9%) | 25 (15.2%) | 19 (22.6%) | 62 (14.5%) |
| Other health challenges | 11 (4.1%) | 10 (5.0%) | 4 (7.8%) | 25 (4.8%) | 1 (0.6%) | 3 (1.8%) | 6 (7.1%) | 10 (2.3%) |
| Finances | 14 (5.6%) | 3 (1.6%) | 2 (4.3%) | 19 (4.0%) | 2 (1.2%) | 5 (3.4%) | 0 (0.0%) | 7 (1.8%) |
| Child care | 17 (6.8%) | 4 (2.2%) | 1 (2.2%) | 22 (4.6%) | 14 (8.2%) | 3 (2.0%) | 1 (1.4%) | 18 (4.6%) |
| Elder care | 2 (0.8%) | 2 (1.1%) | 3 (6.5%) | 7 (1.5%) | 1 (0.6%) | 1 (0.7%) | 1 (1.4%) | 3 (0.8%) |
| Family concerns | 16 (6.4%) | 10 (5.5%) | 0 (0.0%) | 26 (5.4%) | 6 (3.6%) | 4 (2.6%) | 1 (1.4%) | 11 (2.8%) |
