# Supplementary figures and images for: The Masters athlete in Olympic weightlifting: Training, lifestyle, health challenges, and gender differences
Source: PLoS One. 2020 Dec 4;15(12):e0243652. doi: 10.1371/journal.pone.0243652 (PMC7717526; doi:10.1371/journal.pone.0243652)

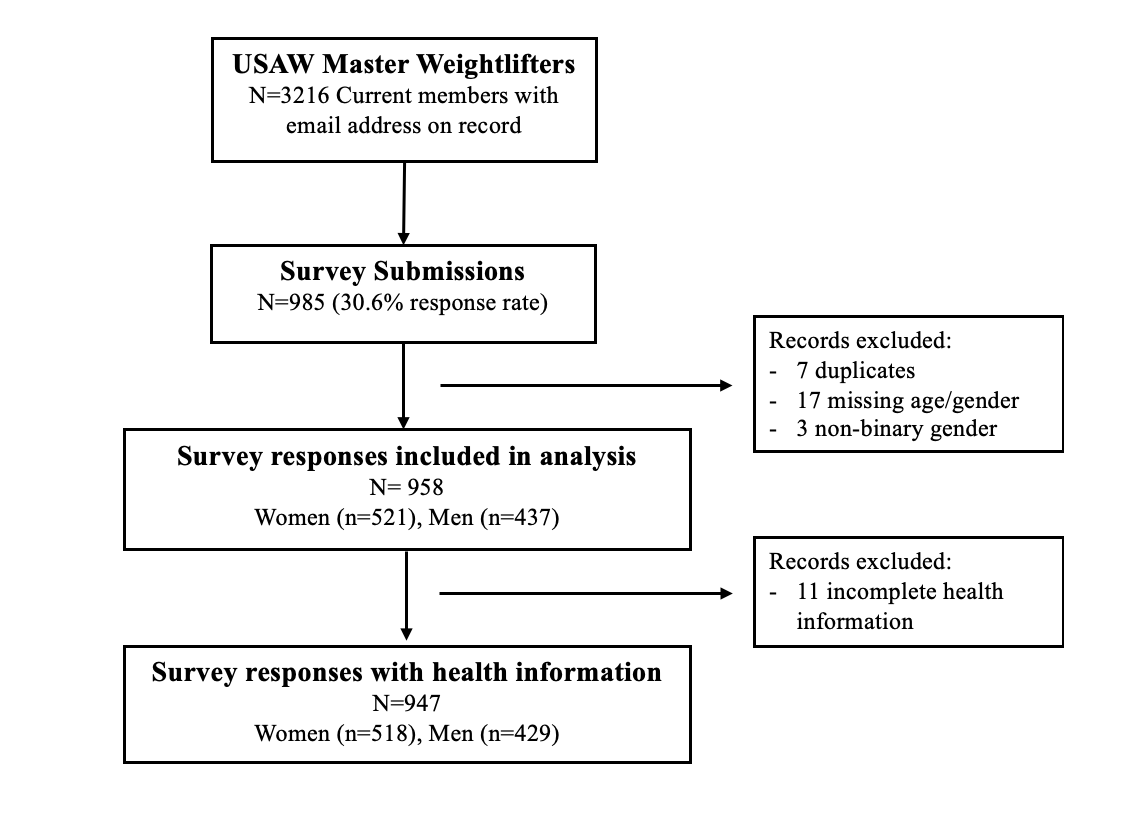

Supplement: S1 Fig — (TIF) [file pone.0243652.s003.tif]

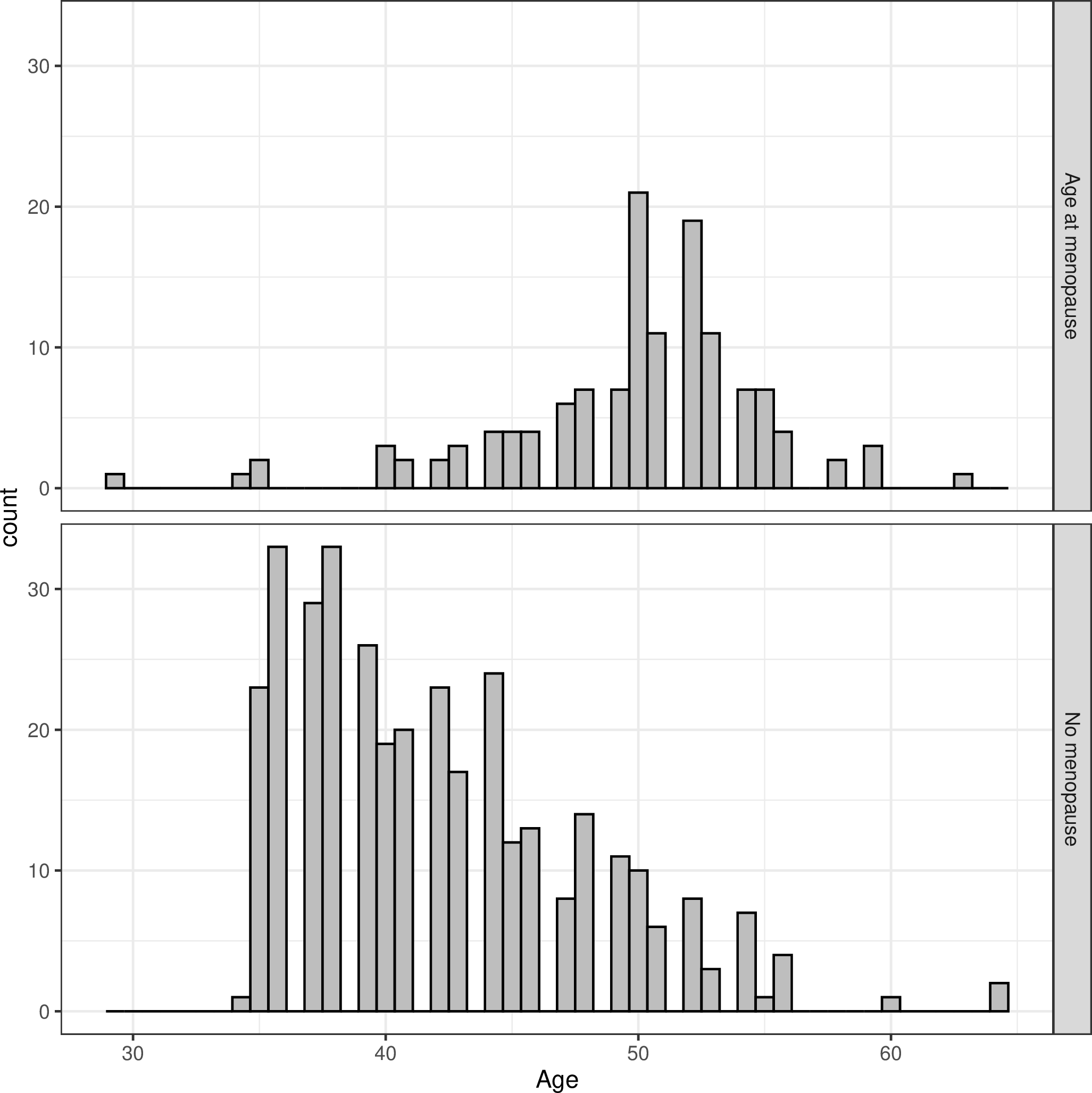

Supplement: S2 Fig — (TIFF) [file pone.0243652.s004.tiff]
