# Supplementary material for: The Masters athlete in Olympic weightlifting: Training, lifestyle, health challenges, and gender differences
Source: PLoS One. 2020 Dec 4;15(12):e0243652. doi: 10.1371/journal.pone.0243652 (PMC7717526; doi:10.1371/journal.pone.0243652)
Supplement: S1 File — (PDF) [file pone.0243652.s005.pdf]

# Survey for Master Weightlifters

## Demographics

**What is the gender that you identify with?**

- ○ Male
- ○ Female
- ○ Transgender
- ○ Intersex
- ○ Other (Please specify) ________________________________________________

**What is your date of birth (Please fill in the format of MM/DD/YYYY)?**

_________________________________________________________________

**Are you Hispanic or Latinx?**

- ○ No
- ○ Yes

**What is your racial identity/identities? Click all that apply.**

- ☐ White/Caucasian
- ☐ Black or African American
- ☐ American Indian or Alaska Native
- ☐ Asian
- ☐ Native Hawaiian or Pacific Islander
- ☐ Other (Please specify) ________________________________________________

**What is your highest level of education you completed?**

- ○ Not graduated from high school
- ○ High school or GED
- ○ Some college
- ○ College degree
- ○ Some graduate school
- ○ Graduate school

**What is your marital status?**

- ○ Unmarried
- ○ Married
- ○ Separated
- ○ Divorced
- ○ Widowed

**What is your employment status?**

- ○ Retired
- ○ Unemployed/Not working
- ○ Part-time employed
- ○ Full-time employed

**What is your employment status?**

- O Retired
- O Unemployed/Not working
- O Part-time employed
- O Full-time employed

**What is your annual household income last year?**

- O $15,000 to $24,999
- O $25,000 to $34,999
- O $35,000 to $49,999
- O $50,000 to $74,999
- O $75,000 to $99,999
- O $100,000 to 149,999
- O $150,000 to $199,999
- O $200,000 and over
- O Decline to answer

## Sports background and participation/Weightlifting

**What was your approximate age when you**

| | Age |
|---|---|
| Started strength training using weights on a regular basis | |
| Started training in Olympic Weightlifting | |

**In which year did you first compete in a sanctioned weightlifting meet? If unsure, give an approximate year.**

____________________________________________________________

**How many different coaches have you had over your weightlifting career (coaches who specifically worked with you specifically on your lifts, e.g. USAW coaches, Crossfit coaches, personal trainers)?**

- O Coach
- O 1 Coach
- O 2 Coaches
- O 3 Coaches
- O More than 3 coaches (Please specify number) ________________________________

**Are you currently working with a weightlifting coach?**

- O No
- O Yes

**If No is selected skip to question “What program(s) do you follow in your weightlifting training?”**

**How do you work with your current coach?**

- ○ In person
- ○ Remotely
- ○ Both

**How frequently does your current coach provide feedback on your weightlifting?**

- ○ Weekly or more often
- ○ Several times each month
- ○ Once a month
- ○ Less frequent than once a month

**What program(s) do you follow in your weightlifting training?**

- ☐ The program assigned by my coach(s)
- ☐ Paid subscription program
- ☐ A program from a website or a book
- ☐ My own program
- ☐ No systematic program
- ☐ Other (Please specify) ________________________________________________

**What is your competition experience with sports other than weightlifting? Please check all that apply.**

| | Competed in the past | | | Competed in the past | | |
|---|---|---|---|---|---|---|
| Crossfit | Never | Occasionally | Often | Never | Occasionally | Often |
| Cardio (Running /Swimming/Cycling) | Never | Occasionally | Often | Never | Occasionally | Often |
| Ball Sports | Never | Occasionally | Often | Never | Occasionally | Often |
| Gymnastics / Cheer leading | Never | Occasionally | Often | Never | Occasionally | Often |
| Martial Arts/Wrestling/ Boxing | Never | Occasionally | Often | Never | Occasionally | Often |
| Track and field | Never | Occasionally | Often | Never | Occasionally | Often |
| Powerlifting | Never | Occasionally | Often | Never | Occasionally | Often |
| Skiing/Snowboarding/Skating | Never | Occasionally | Often | Never | Occasionally | Often |
| Other (Please specify) | Never | Occasionally | Often | Never | Occasionally | Often |

**Currently in a typical week on how many days do you engage in the following physical activities?**

| | 0 day | 1 day | 2 day | 3 day | 4 day | 5 day | 6 day | 7 day |
|---|---|---|---|---|---|---|---|---|
| Weightlifting | ○ | ○ | ○ | ○ | ○ | ○ | ○ | ○ |
| Crossfit | ○ | ○ | ○ | ○ | ○ | ○ | ○ | ○ |
| Cardio (Running /Swimming/Cycling) | ○ | ○ | ○ | ○ | ○ | ○ | ○ | ○ |
| Ball Sports | ○ | ○ | ○ | ○ | ○ | ○ | ○ | ○ |
| Gymnastics / Cheer leading | ○ | ○ | ○ | ○ | ○ | ○ | ○ | ○ |
| Martial Arts/Wrestling/ Boxing | ○ | ○ | ○ | ○ | ○ | ○ | ○ | ○ |
| Track and field | ○ | ○ | ○ | ○ | ○ | ○ | ○ | ○ |
| Walking | ○ | ○ | ○ | ○ | ○ | ○ | ○ | ○ |
| Yoga/Pilates | ○ | ○ | ○ | ○ | ○ | ○ | ○ | ○ |
| Other sport (Please specify) | ○ | ○ | ○ | ○ | ○ | ○ | ○ | ○ |

## Training schedule and Location

**Currently how long does a typical weightlifting training session last for you?**

- O Less than 1 hour
- O About 1 hour
- O 1 to 1.5 hours
- O 1.5 to 2 hours
- O 2 hours or longer

**Where do you typically train for weightlifting (Check all that apply)?**

- O Weightlifting gym
- O Crossfit gym
- O Fitness center
- O Home/garage gym
- O Other (Please specify)________________________________________________

## Health/Mobility/other factors affecting weightlifting training and performance

**In the past two years, overall to what degree have the following factors affected your weightlifting training?**

| | Not at all | Slightly | Moderately | Considerably |
|---|---|---|---|---|
| Work demands | O | O | O | O |
| No access to a training facility | O | O | O | O |
| Sport injuries | O | O | O | O |
| Injuries other than sport injuries | O | O | O | O |
| Surgery | O | O | O | O |
| Pregnancy | O | O | O | O |
| Other health challenges (Please specify) | O | O | O | O |
| Financial difficulties | O | O | O | O |
| Demands of other sports | O | O | O | O |
| Childcare | O | O | O | O |
| Eldercare | O | O | O | O |
| Other family-related issues | O | O | O | O |
| Other challenges (Please specify) | O | O | O | O |

**In the past two years, in total for how long have the following factors interrupted your weightlifting training?**

| | No interruption | Less than 2 weeks | 2 weeks to 1 month | 1 to 6 month | 6 month to 1 year | More than 1 year |
|---|---|---|---|---|---|---|
| Work demands | O | O | O | O | O | O |
| No access to a training facility | O | O | O | O | O | O |
| Sport injuries | O | O | O | O | O | O |
| Injuries other than sport injuries | O | O | O | O | O | O |
| Surgery | O | O | O | O | O | O |
| Pregnancy | O | O | O | O | O | O |
| Other health challenges (Please specify) | O | O | O | O | O | O |
| Financial difficulties | O | O | O | O | O | O |
| Demands of other sports | O | O | O | O | O | O |
| Childcare | O | O | O | O | O | O |
| Eldercare | O | O | O | O | O | O |
| Other family-related issues | O | O | O | O | O | O |
| Other challenges (Please specify) | O | O | O | O | O | O |

**In the past two years, to what degree did any of the following physical or psychological issues constrain/restrict your training?**

| | Not at all | Slightly | Moderately | Considerably |
|---|---|---|---|---|
| Shoulders | O | O | O | O |
| Back | O | O | O | O |
| Hips | O | O | O | O |
| Knees | O | O | O | O |
| Other joints | O | O | O | O |
| Anxiety | O | O | O | O |
| Depression | O | O | O | O |
| Stress | O | O | O | O |
| Trouble sleeping | O | O | O | O |
| Cardiovascular | O | O | O | O |
| Cancer | O | O | O | O |
| Diabetes | O | O | O | O |
| Autoimmune diseases | O | O | O | O |
| Respiratory illnesses and diseases | O | O | O | O |
| Digestive disorder and Gastrointestinal diseases | O | O | O | O |
| Other chronic health issues | O | O | O | O |
| Menstruation | O | O | O | O |
| Peri/Menopause symptoms | O | O | O | O |

**Display The question "Have you experienced menopause (no menstruation for a year)?" if selected "Female", or "Transgender", or "Intersex", or "Other" in question "What is the gender that you identify with?"**

**Have you experienced menopause (no menstruation for a year)?**

- ○ No
- ○ Yes
- ○ Not applicable

**Display Question "Please tell us the age when you reached menopause (no menstruation for a year)" if select Yes for question "Have you experienced menopause (no menstruation for a year)?**

**Please tell us the age when you reached menopause (no menstruation for a year).**

________________________________________________________________

**Display Question "Which peri/menopausal symptoms have disrupted your training or competing in weightlifting? Please check all that apply." if select Yes for question "Have you experienced menopause (no menstruation for a year)?**

**Which peri/menopausal symptoms have disrupted your training or competing in weightlifting? Please check all that apply.**

- ☐ Hot flashes
- ☐ Fatigue
- ☐ Trouble sleeping
- ☐ Mood swings
- ☐ Weight gain and slowed metabolism
- ☐ Other (18) ________________________________________________

## Comments

Your responses will help us better understand the factors that affect masters weightlifting performance, and how this varies for different individuals.

**With your permission, we would like to connect the responses of this questionnaire to your performance trajectories. If you are willing, please provide your USAW number below.**

- ○ Yes (Please provide your USAW number)
- ○ No

**Do you have any additional comments about the factors affecting your weightlifting career?**

________________________________________________________________

**Do you have any comments about how your weightlifting career affects parts of your life?**

________________________________________________________________
